# Patient-specific guided Deep Circumflex Iliac Artery free flap for mandibular reconstruction in an ameloblastoma case. Case report and insights on the current state of customized surgery in H&N reconstruction in China

**DOI:** 10.4317/jced.62693

**Published:** 2025-04-01

**Authors:** Íñigo Aragón Niño, Chongyang Zheng, Han Cheng, Yongjie Hu, Yue He

**Affiliations:** 1 Oral and Maxillofacial - Head & Neck Oncology Department. Ninth People´s Hospital. Shanghai, China; 2 IdiPAZ Translational Research Group in OMFS and H&N Cancer. La Paz University Hospital. Madrid, Spain

## Abstract

This article presents a case of recurrent mandibular ameloblastoma treated with customized reconstructive surgery using a microvascularized DCIA bone flap. Virtual surgical planning and customized surgery, which has become increasingly adopted in head and neck reconstruction, enhances precision in tumor resection, flap design, and fixation, leading to superior functional and aesthetic outcomes. The patient, initially treated with curettage in China (2017) and Japan (2021), presented with recurrence upon relocating to Shanghai. Imaging confirmed the diagnosis, and customized surgery was planned due to the complexity of the case. The surgical approach involved patient-specific cutting guides for both the mandibular resection and iliac crest flap harvesting, ensuring precise bone segmentation and reconstruction. Contouring guides were used to optimize bone alignment, and a pre-bent titanium reconstruction plate was employed. Despite the advantages of customized surgery, its widespread implementation in China faces regulatory and financial challenges. Restrictions on patient-specific implants and high costs limit accessibility, but alternative techniques, such as contouring guides, help improve outcomes. As regulations evolve and technology advances, customized surgery is expected to become a standard approach, improving reconstructive care in complex cases.

** Key words:**Mandibular ameloblastoma, guided surgery, customized reconstruction, head and neck, surgery in China.

## Introduction

This article describes the use of customized surgery for a case of recurrent mandibular ameloblastoma in a patient who underwent reconstruction using a microvascularized DCIA bone flap.

Head and neck reconstructive surgery has evolved in recent years toward customized, patient-specific solutions tailored to each case. In the last decade, the adoption of virtual surgical planning and customized reconstructive surgery has increased globally and is likely to become the standard of care in the near future (1-3).

Customized surgery involves the use of patient-specific solutions adapted to the particular clinical scenario. Each case is planned in advance, defining the surgical steps including tumor resection, flap harvesting, and fixation. Based on this, cutting guides for resection, flap harvesting guides, and positioning guides for inset into the recipient site are designed. Additionally, if necessary, a custom-made titanium reconstruction plate is fabricated, adapted to the new bone contour and the properly positioned flap.

However, the implementation of these solutions is not uniform across all countries. In China, local regulations do not allow the fabrication of patient-specific plates, making importation the only alternative, which is often restricted or simply unfeasible due to high costs. Furthermore, the public healthcare system operates on a co-payment basis, meaning that patients contribute to the cost of treatment. This financial model favors the use of the most cost-effective and efficient solutions, often relying on local companies.

Mandibular ameloblastoma is a benign but locally aggressive odontogenic tumor originating from the enamel epithelium. It accounts for approximately 1% of oral tumors and 10–15% of odontogenic tumors. It most commonly affects the mandible, particularly the posterior region, and is typically diagnosed in young adults, with a slight male predominance. Clinically, it may be asymptomatic or present with bone expansion, pain, and dental mobility. Radiographically, it often appears as a multilocular lesion with a “honeycomb” pattern. Due to its high recurrence rate following conservative treatment, surgical resection remains the mainstay of management.

Case Report

This article presents the case of a patient diagnosed with mandibular ameloblastoma who underwent curettage at the Affiliated Stomatological Hospital of Harbin Medical University (China) in 2017. After a four-year postoperative follow-up, recurrence was detected, leading to a second curettage at the Affiliated Hospital of Kochi University (Japan) in 2021. The patient remained under regular follow-up until August 2023.

Upon relocating to Shanghai, a follow-up examination at our hospital revealed a cystic-solid lesion involving the symphysis and right mandibular body. Due to ongoing breastfeeding, surgical intervention was initially deferred. Two months prior to admission, the patient developed an unexplained lingual swelling in the anterior mandibular region, without associated pain or discomfort, prompting her to seek medical attention at our institution. Imaging studies confirmed the diagnosis of “recurrent mandibular ameloblastoma following multiple surgical treatments at external institutions,” and the patient was admitted for further evaluation and management.

After completing the necessary preoperative evaluations, surgery was indicated for the patient, with a customized surgical approach planned due to the complexity of the case and the need for a precise and safe treatment strategy. The case design and virtual surgical planning were carried out in collaboration with the local company Shanghai Huifeng Dental Technology Co., Ltd. (Shanghai, China).

For the mandibular lesion resection, a patient-specific cutting guide was used. Additionally, a 3D-printed model of the mandible was prepared preoperatively to facilitate and ensure the accurate positioning of the guide, which was secured to the mandible using three titanium screws on each side. Notably, the positioning of these screws was planned to align with the fixation points of the reconstruction plate to be used in the subsequent phase of the procedure. The entire planning process was designed to optimize screw placement, ensuring that each drilled hole would be reused in the following step, thereby minimizing unnecessary bone perforations.

Once the cutting guide was securely fixed to the mandible, a segmental mandibulectomy from tooth 33 to 47 was performed using a reciprocating saw. The saw was guided along the designated edge of the cutting guide, ensuring that the resection was carried out at the exact preplanned location and with the desired surgical margins, providing high precision.

**Figure 1:**
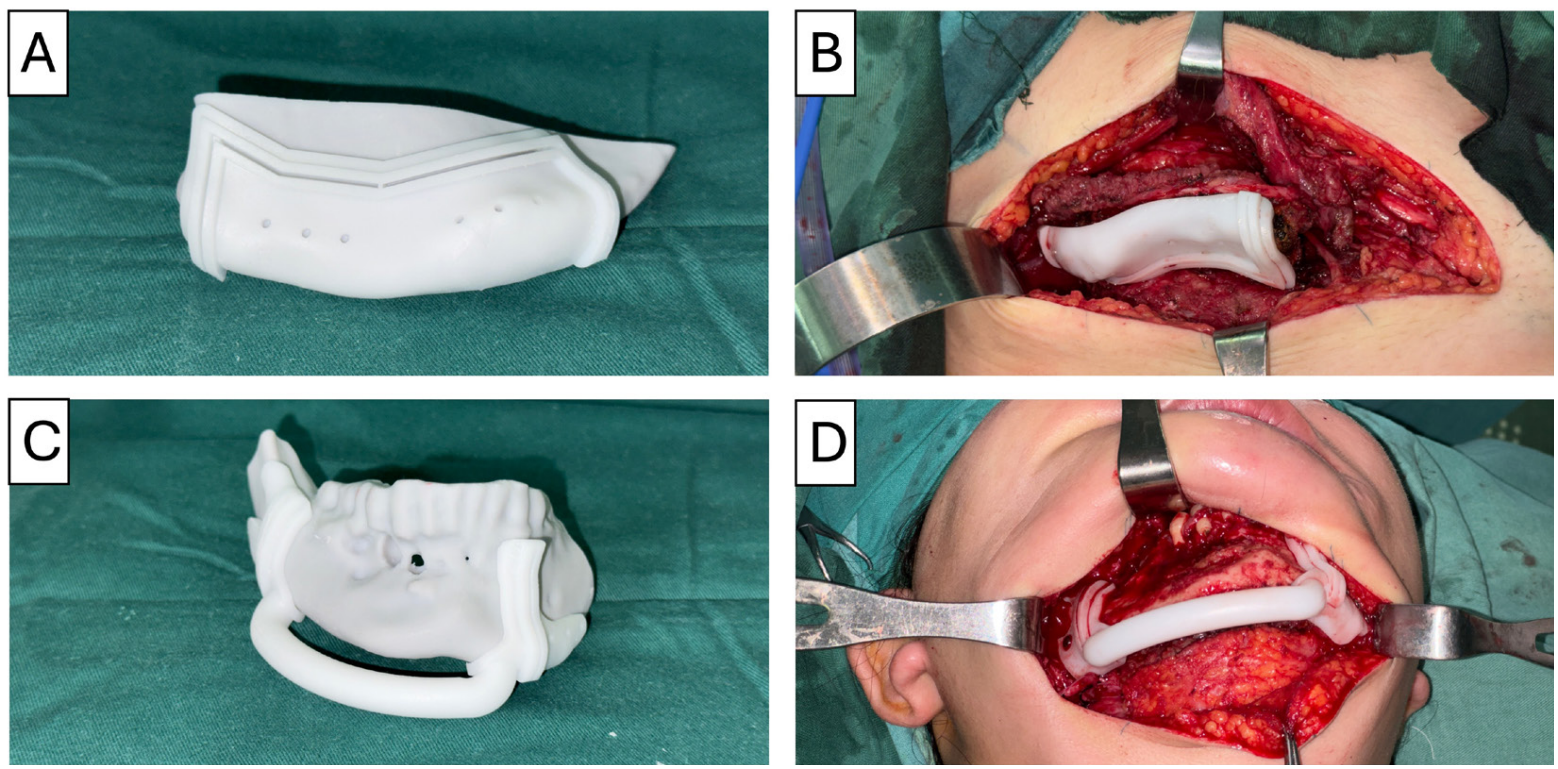
(A) Cutting guide for lesion resection placed on the model. (B) Cutting guide positioned on the patient’s iliac bone. (C) Cutting guide for flap harvesting placed on the model. (D) Cutting guide positioned on the patient’s mandible.

Simultaneously, in a separate surgical field, an approach to the right iliac crest was performed for harvesting the myo-osseous DCIA bone flap. Once the flap was exposed, a patient-specific cutting guide was used to define the osteotomies. The cuts were then executed using a reciprocating saw, following the guide’s designated slots to ensure accurate bone segmentation (Fig. 1).

In this case, the reconstruction required a two-segment bone flap. Therefore, two independent cutting guides were designed to separate the segments, labeled A and B.

**Figure 2:**
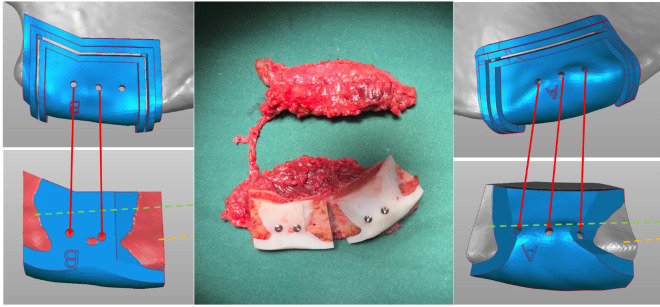
Use of contouring guides in the surgical plan and applied to the iliac crest bone flap.

The next step involved the use of contouring guides, which were applied both to the mandible and to the bone segments of the reconstruction flap. This intermediate step added precision to the process, enhancing the accuracy of the final reconstruction (Fig. 2).

**Figure 3:**
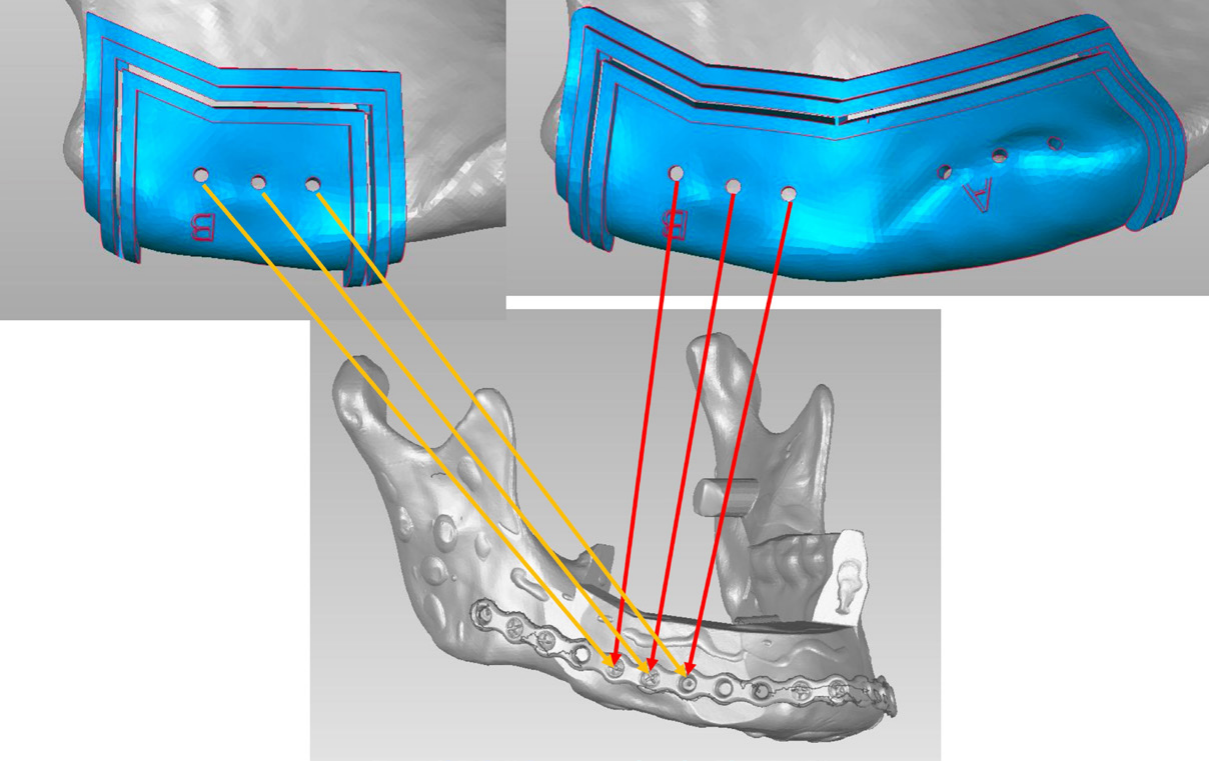
Planning for the use of the same screw positions across all phases of surgery.

The screws used for securing the initial cutting guide, the flap segmentation guide, and the contouring guides were all pre-planned to align with the future screw positions of the reconstruction plate (Fig. 3).

**Figure 4:**
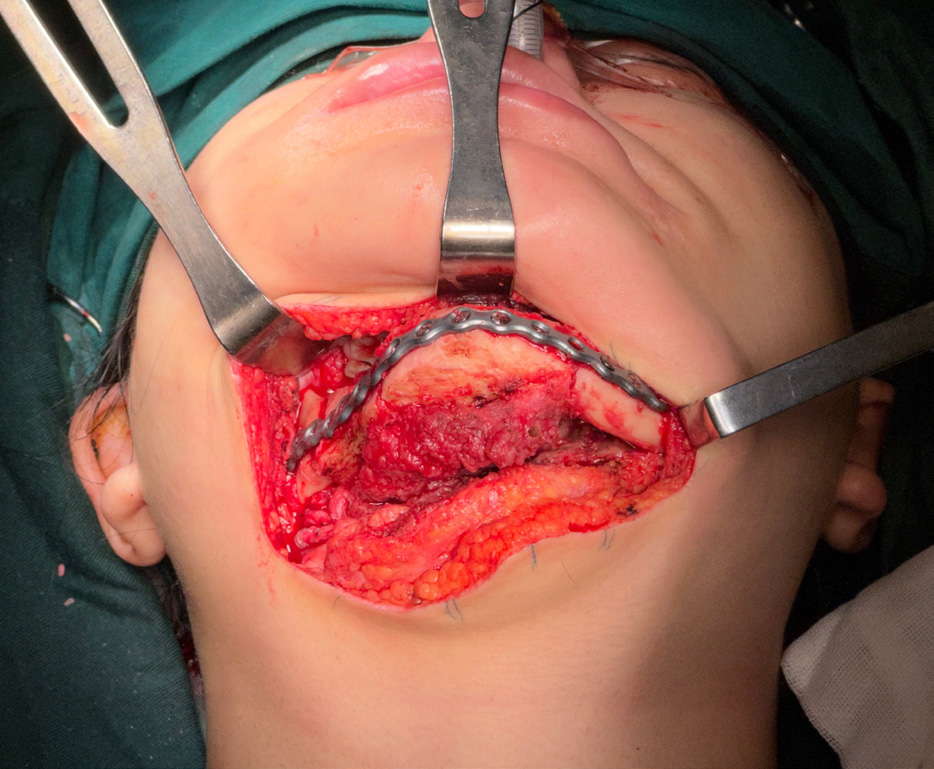
Final result of the reconstruction. Following fixation, microsurgical anastomosis was performed to the ipsilateral facial artery and external jugular vein. The procedure was concluded with the placement of surgical drains and the closure of incisions and the surgical site.

The final step involved flap inset and fixation to the mandible using a pre-shaped 2.5 mm titanium reconstruction plate, which had been pre-molded according to the patient’s final reconstruction model. It is important to note that in China, the fabrication and use of IPS titanium plates are not approved. Consequently, this alternative pre-molding solution provided by the manufacturer was employed (Fig. 4).

## Discussion

Reconstructive surgery is evolving across all fields, but its advancements are particularly noTable in head and neck reconstructive surgery. In this anatomical region, precision is paramount, not only in resection, where removing too little tissue could result in suboptimal surgery with inadequate margins, but also in avoiding excessive resection, which could lead to significant aesthetic implications for patients. Additionally, the reconstructive phase is crucial for ensuring optimal functional and aesthetic outcomes, given the high esthetic standards required in facial surgery. These factors have driven the development of customized reconstructive surgery, tailored to each patient and their specific case (1-3).

The advantages of this approach include more precise resections that align with preoperative planning and oncologic margins, a flap design that perfectly matches the planned defect, and optimal adaptation to the recipient site. This leads to improved surgical outcomes, enhanced precision, and superior aesthetic results (4-7).

In this case, the lesion is benign but locally aggressive, with a high recurrence rate, affecting a young patient. Therefore, customized surgery is particularly indicated, as it allows for a precise resection that maximizes the likelihood of complete tumor removal while minimizing unnecessary tissue loss. Simultaneously, it facilitates reconstruction with excellent aesthetic results, which is essential in such cases.

The widespread implementation of customized surgery in China is still an ongoing process due to two main challenges. First, there are regulatory restrictions on the fabrication of patient-specific surgical devices, as the legal framework in this area is still under development, and such devices have not yet been approved. Second, the high cost of these procedures compared to conventional alternatives, combined with the healthcare system’s co-payment model, where patients bear part of the financial burden, limits their accessibility. Despite these challenges, the desire to advance customized surgery has persisted, leading to adaptations where possible. One such example is the use of contouring guides for both the resection site and the reconstruction flap. A common limitation in these reconstructions is that, despite meticulous preoperative planning, minor inaccuracies at the junctions of bone segments during surgery can prevent a seamless fit. Contouring guides aim to address this issue by ensuring a precise bone-to-bone fit, improving the final outcome of the reconstruction.

Customized reconstructive surgery represents a significant advancement in head and neck surgery, offering superior precision, improved functional and aesthetic outcomes, and better adaptation to individual patient needs. Despite regulatory and financial challenges limiting its widespread adoption in China, ongoing efforts to implement customized techniques, such as contouring guides, demonstrate the potential for overcoming these barriers. As technology and regulations evolve, the integration of patient-specific solutions is expected to become more accessible, ultimately improving the standard of care in complex reconstructive cases.

## Data Availability

The datasets used and/or analyzed during the current study are available from the corresponding author.
